# The role of antiplatelet therapies on incidence and mortality of hepatocellular carcinoma

**DOI:** 10.1111/eci.13870

**Published:** 2022-09-16

**Authors:** Quirino Lai, Nicoletta De Matthaeis, Michele Finotti, Giovanni Galati, Giuseppe Marrone, Fabio Melandro, Filomena Morisco, Daniele Nicolini, Riccardo Pravisani, Edoardo G. Giannini, the Associazione Italiana per lo Studio del Fegato (AISF) HCC Special Interest Group, Aglitti A, Aliberti C, Baccarani U, Bhoori S, Borzio M, Brancaccio G, Burra P, Cabibbo G, Casadei Gardini A, Carrai P, Cillo U, Conti F, Cucchetti A, D’Ambrosio R, Dell’Unto C, Di Costanzo GG, Di Sandro S, Foschi FG, Fucilli F, Gambato M, Gasbarrini A, Giuliante F, Ghinolfi D, Grieco A, Gruttaduria S, Guarino M, Kostandini A, Iavarone M, Lenci I, Levi Sandri GB, Losito F, Lupo LG, Manzia TM, Mazzocato S, Mescoli C, Miele L, Muley M, Persico M, Plaz Torres MC, Pompili M, Ponziani FR, Rapaccini GL, Rendina M, Renzulli M, Rossi M, Rreka E, Russo FP, Sacco R, Sangiovanni A, Sessa A, Simonetti N, Sposito C, Tortora R, Trevisani F, Viganò L, Viganò M, Villa E, Vincenzi V, Violi P, Vitale A

**Affiliations:** ^1^ General Surgery and Organ Transplantation Unit AOU Policlinico Umberto I, Sapienza University of Rome Rome Italy; ^2^ Department of Medical and Surgical Sciences, Fondazione Policlinico Gemelli Istituto di Ricovero e Cura a Carattere Scientifico (IRCCS) Rome Italy; ^3^ 4th Surgery Unit, Regional Hospital Treviso, DISCOG University of Padua Padua Italy; ^4^ Unit of Clinical Medicine and Hepatology University Campus Bio‐Medico Rome Italy; ^5^ Hepatobiliary surgery and liver transplantation University of Pisa Medical School Hospital Pisa Italy; ^6^ Department of Clinical Medicine and Surgery, Gastroenterology and Hepatology Unit University of Naples "Federico II" Napoli Italy; ^7^ Unit of Hepatobiliary Surgery and Transplantation, Polytechnic University of Marche Azienda Ospedaliero‐Universitaria "Ospedali Riuniti" Torrette Ancona Italy; ^8^ Liver‐Kidney Transplantation Unit, Department of Medicine University of Udine Udine Italy; ^9^ Gastroenterology Unit, Department of Internal Medicine, IRCCS Ospedale Policlinico San Martino University of Genoa Genoa Italy

**Keywords:** aspirin, clopidogrel, incidence, occurrence, survival

## Abstract

**Aim:**

To evaluate the impact of antiplatelet therapy (APT)on the incidence of hepatocellular carcinoma (HCC) and mortality following its treatment.

**Methods:**

A systematic literature search was performed using PubMed and Cochrane Central Register of Controlled Trials Databases. Two HCC clinical settings were explored: (i) incidence, and (ii) death after any HCC treatment. Odds ratios (OR) and 95% confidence intervals (95%CI) were calculated to compare the pooled data between patients who received or did not receive APT.

**Results:**

A total of 20 studies were identified, of whom 15 focused on HCC incidence, including 2,685,009 patients, and five on post‐treatment death, including 3281 patients. APT was associated with an overall reduced risk of HCC incidence (OR: 0.63; 95%CI = 0.51–0.79; *p* < 0.001) as well as of post‐treatment mortality (OR: 0.54; 95%CI = 0.35–0.83; *p* = 0.006).

**Conclusions:**

Current data suggest that APT correlated with higher HCC incidence and poor overall survival following tumour treatment.

**Abbreviations**
APT: anti‐platelet therapies
CIs: confidence intervals
COX‐1: cyclooxygenase‐1
EGF: Epidermal Growth Factor
HCC: hepatocellular carcinoma
I2: Higgins statistic squared
NOS: Newcastle‐Ottawa Quality Assessment Scale
NSAIDs: non‐steroidal anti‐inflammatory drugs
OR: odds ratio
PDGF: Platelet Derived Growth Factor
PICO: Patients, Intervention, Comparator, Outcome
PRISMA: Preferred Reporting Items for Systemic Reviews and Meta‐Analysis
TGF‐β: Transforming Growth Factor‐β
VEGF: Vascular Endothelial Growth Factor

## 1 INTRODUCTION

In the last decades, the complex relationship between platelets and hepatocellular carcinoma (HCC) has been largely studied, both in vitro and in vivo, with results showing that platelets can be considered a central player in liver regeneration and hepatocarcinogenesis, with several platelet‐related factors such as serotonin, platelet‐derived growth factor (PDGF),epidermal growth factor (EGF) and vascular endothelial growth factor (VEGF) variously acting in the angiogenic, inflammatory and proliferative processes.^1^, ^2^ Furthermore, platelets are also involved in tumour spread, as they may favour the permeabilization of the endothelium, thus allowing the passage of neoplastic cells into the bloodstream.^3^

While the biologic mechanisms underlying the role played by platelets on HCC formation and progression have been well described, the potential role of antiplatelet therapies (APT) on the interaction between HCC and the host remains poorly explored. Indeed, while the benefits of aspirin for the prevention of colorectal cancer are well‐established, a growing body of evidence suggests that aspirin and clopidogrel may also help prevent HCC through several biological mechanisms.^4^, ^5^, ^6^, ^7^ In detail, aspirin blocks the release of both serotonin and cyclooxygenase‐1 (COX‐1), with consequent suppression of thromboxane‐A2, which promotes a pro‐metastatic *niche* involving both endothelial and tumour cells.^8^, ^9^, ^10^ Conversely, clopidogrel inhibits the expression of α‐granule‐stored proteins (i.e. P‐selectin and CD40L), playing a crucial role in the passage from vascular injury to inflammation; these proteins are involved in heterotypic interactions between platelets/leukocytes and the endothelium ^8^, ^11^. Moreover, according to a recent study based on a murine model, the clopidogrel P2Y12‐inhibitor effect decreases the number of tumoral cells by promoting an anti‐tumoral macrophage phenotype.^12^ Therefore, there is compelling initial evidence that APT may modulate the interaction between HCC and the host, with potentially positive effects.

Thus, to gain a better insight into this issue, we performed a meta‐analysis to evaluate the impact of APT on the risk of HCC incidence and mortality following its treatment. To this end, we focused on two different events of interest, namely HCC incidence and death after any type of HCC therapy.

## 2 MATERIALS AND METHODS

### 2.1 Search methodology and study design

A systematic review of the published literature was carried out, focusing on the role of APT in the incidence of HCC and patient mortality after HCC treatment. The search strategy followed the Preferred Reporting Items for Systemic Reviews and Meta‐analysis (PRISMA) guidelines.^13^

A search of the PubMed and Cochrane Central Register of Controlled Trials Databases was conducted using the following terms: (anti platelet OR antiplatelet OR APT OR DAPT OR dual antiplatelet OR dual anti platelet OR aspirin OR clopidogrel OR ticlopidine OR dipyridamole OR thromboxane inhib*) AND (HCC OR hepatoma OR hepatocell*). The search period was from ‘2000/01/01’ to ‘2022/03/31’. The systematic qualitative review included only studies in English that included humans. Published reports were excluded based on several criteria: (a) data on animal models alone; (b) lack of enough clinical details; (c) use of non‐primary source data (e.g. review articles, non‐clinical studies, letters to the editor, expert opinions and conference summaries). In the case of studies originating from the same centre, the potential overlap of clinical cases was examined, and the most informative study was considered eligible.

Two different specific research questions were formulated with this research.

For the incidence of HCC, the Patients, Intervention, Comparator, Outcome (PICO) components were:

- Patient: patient with APT;
- Intervention: follow‐up;
- Comparison: patient without APT receiving the same follow‐up;
- Outcome: HCC incidence.

While for the outcome following HCC treatment, the PICO components were:

- Patient: patient with HCC and APT;
- Intervention: any HCC therapy;
- Comparison: patient with HCC without APT treated with the same approach;
- Outcome: death.

### 2.2 Data extraction and definitions

Following a full‐text review of the eligible studies, two independent authors (QL and FM) performed the data extraction and cross‐checked all outcomes. During the articles selection and data extraction process, potential discrepancies were resolved following a consensus with a third reviewer (GM). Collected data included the first author of the publication, year of publication, country, the study period, the type of ATP used, the total number of cases, the number of events/treated cases in the ATP and no‐ATP groups, the indication for APT use, the duration of the APT therapy, age, sex, presence of cirrhosis and study follow‐up time.

### 2.3 Quality assessment

Selected studies were systematically reviewed with the intent to identify potential sources of bias. The quality of the published studies was assessed using the Newcastle‐Ottawa Quality Assessment Scale (NOS). Studies with a score >6 were defined as high‐quality studies.^14^

### 2.4 Statistical analysis

Study results are expressed as odds ratio (OR) and 95% confidence intervals (95%CIs). The statistical heterogeneity was evaluated with the Higgins statistic squared (I2). I2 values of 0%–25% were considered as an index of low heterogeneity between studies, 26%–50%: moderate heterogeneity, and ≥ 51%: high heterogeneity. The fixed‐effects model was used when low or moderate (0%–50%) heterogeneity was detected between studies, while the random effects model was preferred when high heterogeneity was present. A *p*‐value<0.05 was considered indicative of statistical significance. The meta‐analysis was performed using OpenMetaAnalyst (http://www.cebm.brown.edu/openmeta/index.html).

## 3 RESULTS

### 3.1 Search results and study characteristics

**FIGURE 1:**
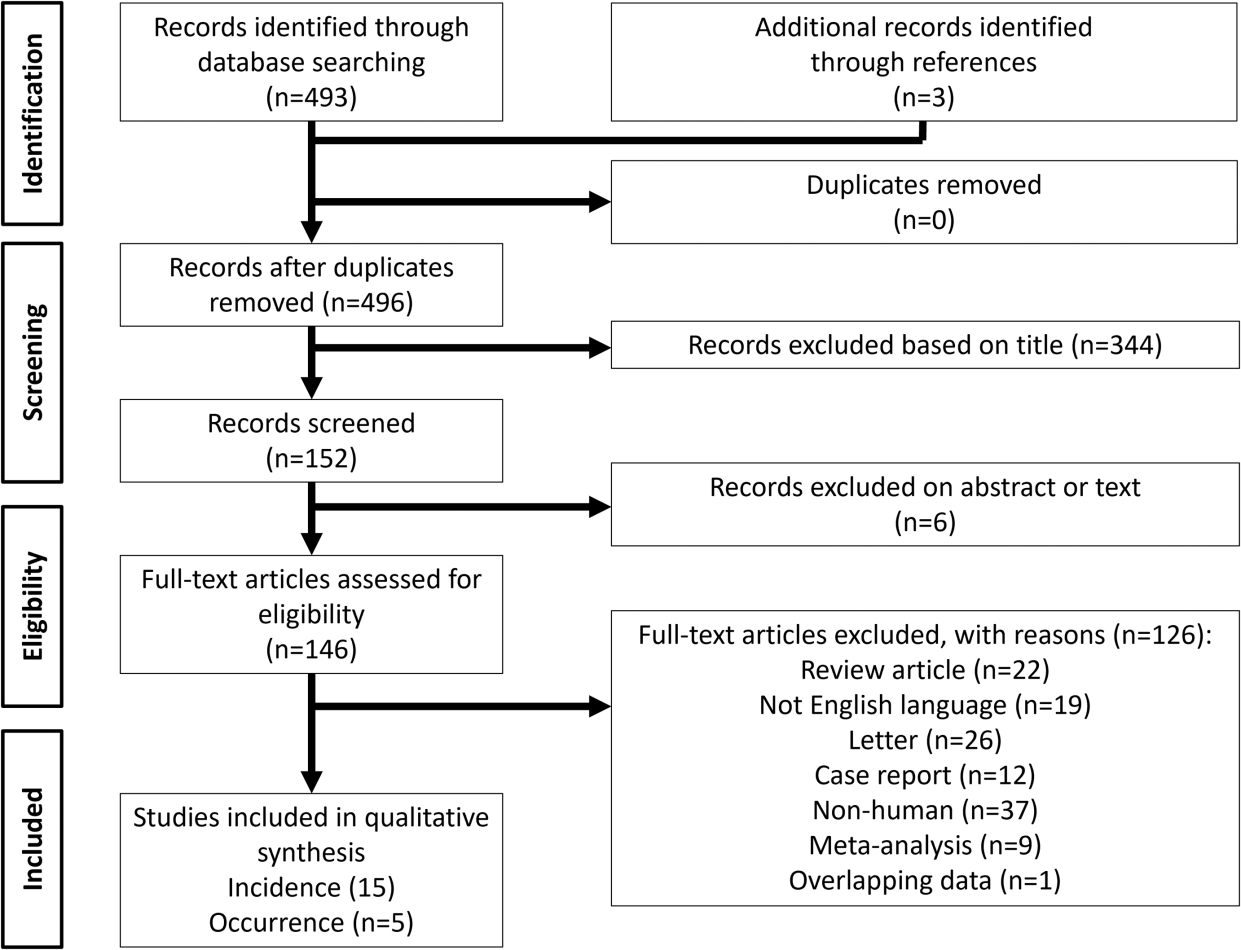
PRISMA summarizing the trial flows for HCC incidence and occurrence

The PRISMA flow diagram depicts the article selection process (Figure 1). Briefly, among the 493 articles screened, 156 articles were assessed for eligibility with 20 studies, 15 on HCC incidence and five on mortality outcome following treatment, which were finally included in the meta‐analysis.^15^, ^16^, ^17^, ^18^, ^19^, ^20^, ^21^, ^22^, ^23^, ^24^, ^25^, ^26^, ^27^, ^28^, ^29^, ^30^, ^31^, ^32^, ^33^, ^34^

#### 3.1.1 Qualitative assessment of the included studies

Using the NOS tool quality assessment, 13/15 studies (86.7%) had a score >6 and were therefore considered high‐quality studies (Table 1).

**TABLE 1:** Characteristics of included studies for the risk of HCC incidence and outcome following HCC treatment

Ref.	Name	Year	Country	Study period	Design study	APT type	N	APT	Events	No APT	Events	NOS
HCC incidence												
29	Jang H	2022	Korea	2007–2017	Retro	Asp	35,309	17,771	1232	17,538	1465	9
28	Singh J	2021	US	2012–2017	Retro	Asp ‐ Clo	521	170	6	351	39	7
27	Hui VWK	2021	Hong Kong	2000–2018	Retro	Asp ‐ Ibu	35,111	1744	69	33,367	1488	9
26	Choi WM	2021	Korea	2005–2015	Retro	Asp ‐ NSAID	32,695	7718	1323	24,977	5216	9
25	Simon TJ	2020	Sweden	2005–2015	Retro	Asp	50,275	14,205	338	36,070	1274	9
24	Liao YH	2020	Taiwan	2000–2012	Retro	Asp	3822	1911	131	1911	147	9
23	Lee TY	2020	Taiwan	1997–2011	Retro	Asp	7434	2478	166	4956	270	9
22	Tsoi KKF	2019	Hong Kong	2000–2013	Prosp	Asp	612,509	204,170	1984	408,339	7386	9
21	Simon TJ^2^	2019	US	1980–2012	Prosp	Asp	133,371	58,855	37	74,516	71	9
20	Lee TY^2^	2019	Taiwan	1997–2012	Retro	Asp	10,615	2123	123	8492	574	8
19	Hwang IC	2018	Korea	2002–2006	Prosp	Asp	150,496	75,248	279	75,248	294	9
18	Lee M	2017	Korea	2002–2015	Retro	Asp	840	420	15	420	48	9
17	Petrick JL	2015	US	2009–2015	Prosp	Asp	1,084,133	477,470	315	606,663	364	8
16	Jacobs EJ	2012	US	1997–2018	Prosp	Asp	100,139	76,270	15	23,869	22	8
15	Sahasrabuddhe V	2012	US	1995–1996	Prosp	Asp ‐ NSAID	427,739	387,790	295	39,949	202	8
Post‐HCC treatment death												
34	Young SH	2020	Taiwan	2007–2014	Retro	Asp	430	15	2	415	87	6
33	Boas FE	2019	USA	2009–2016	Retro	Asp	304	42	7	262	127	7
32	Rungsakulkij N	2018	Thailand	2006–2015	Retro	Dual Asp+Tic	217	18	2	199	36	6
31	Lee PC	2016	Taiwan	1997–2011	Retro	Asp ‐ Clo	2210	442	94	1768	512	8
30	Li JH	2016	China	2008–2013	Retro	Asp	120	60	29	60	34	7

Abbreviations: APT, antiplatelet therapy; Asp, aspirin; Clo, clopidogrel; Ibu, ibuprofen; N, number; Newcastle‐Ottawa Score; NSAID, non‐steroidal anti‐inflammatory drugs; Tic, ticlopidine.

#### 3.1.2 Review of the eligible studies

Data extracted from the selected articles are reported in detail in Tables 1 and 2. Among the 15 studies investigating the risk of HCC incidence, the majority (*n* = 9, 60.0%) were carried out in Eastern countries, 5 (33.3%) in the United States and only 1 (6.7%) in Europe.^15^, ^16^, ^17^, ^18^, ^19^, ^20^, ^21^, ^22^, ^23^, ^24^, ^25^, ^26^, ^27^, ^28^, ^29^ Nine studies were based on retrospective databases, while six studies were based on prospective data. No study was randomized controlled trial.

**TABLE 2:** Demographic data of the included studies for the risk of HCC incidence and post‐HCC treatment death

Ref.	Author	Reason for APT	Duration of APT	Age years	Male sex %	Cirrhosis %	FU years
HCC incidence							
29	Jang H	NA	Median time 39 months	No APT 54 (48–62) APT 55 (49–61)	No APT 76 APT 75	No APT 13 APT 13	No APT 6.3 APT 6.4
28	Singh J	NA	NA	59 ± 11	55	100	7
27	Hui VWK	Specified	Median time 34 (19–59) months	No APT 53 ± 13 APT 62 ± 11	No APT 61 APT 469	No APT 7 APT 11	No APT 3.3 APT 2.8
26	Choi WM	NA	Median time 18 (5–39) months	No APT 49 ± 11 APT 54 ± 10	No APT 50 APT 79	NA	No APT 4.9 APT 4.3
25	Simon TJ	NA	3mo–1 year ‐ 1–3 years ‐ 3–5 years ‐ ≥5 years	No APT 40 APT 51	No APT 64 APT 72	No APT 14 APT 14	8
24	Liao YH	NA	1 year ‐ 1–2 years ‐ 2–3 years ‐ ≥3 years	No APT 65 ± 14 APT 64 ± 14	No APT 46 APT 48	No APT 2 APT 1	No APT 4 APT 4
23	Lee TY	NA	Median time 4 (2–7) years	No APT 63 ± 10 APT 63 ± 10	No APT 44 APT 44	No APT 15 APT 16	5
22	Tsoi KKF	Specified	Mean time 92 ± 53 months	68	No APT 54 APT 54	NA	8.9 (3.5–12.7)
21	Simon TJ^2^	NA	<5 years ‐ 5–10 years ‐ ≥10 years	No APT 62 ± 8 APT 64 ± 8	No APT 32 APT 38	NA	26
20	Lee TY^2^	Specified	Median time 37 (14–72) months	No APT 59 ± 12 APT 59 ± 12	No APT 72 APT 72	17	5
19	Hwang IC	NA	Median time 13 (4–29) months	No APT 49 (44–57) APT 58 (50–65)	No APT 54 APT 51	16	6.4
18	Lee M	Specified	Mean time 28 months	No APT 50 ± 11 APT 55 ± 11	No APT 64 APT 61	No APT 12 APT 12	5
17	Petrick JL	NA	<5 years ‐ ≥5 years	No APT 53 ± 13 APT 62 ± 11	No APT 27 APT 48	NA	11.9
16	Jacobs EJ	NA	<5 years ‐ ≥5 years	60	44	NA	16
15	Sahasrabuddhe V	NA	NA	63 ± 5	58	NA	6.4
Post‐HCC treatment death							
34	Young SH	NA	NA	No APT 58 ± 12 APT 64 ± 10	No APT 85 APT 83	No APT 38 APT 23	No APT 4.1 APT 4.8
33	Boas FE	NA	NA	69	76	NA	NA
32	Rungsakulkij N	NA	NA	56 ± 10	46	NA	3.0 (0–12.4)
31	Lee PC	NA	NA	No APT 53 ± 12 APT 63 ± 10	No APT 83 APT 84	No APT 53 APT 50	No APT 3.9 ± 3.2 APT 4.3 ± 3.0
30	Li JH	NA	NA	No APT 66 ± 16 APT 67 ± 16	No APT 80 APT 80	No APT 80 APT 82	25.4 (4.0–82.2)

Abbreviations: APT, antiplatelet therapy; Asp, aspirin; Clo, clopidogrel; Ibu, ibuprofen; N, number; Newcastle‐Ottawa Score; NSAID, non‐steroidal anti‐inflammatory drugs.

Of the five studies that assessed mortality following any HCC treatment, four originated in Eastern countries (80.0%) and one from the United States (20%).^30^, ^31^, ^32^, ^33^, ^34^ All the studies focused on post‐treatment mortality were retrospective studies.

Overall, 13/15 studies on HCC incidence (86.7%) and 1/5 studies on mortality following HCC treatment (20%) were based on cohorts including more than 1000 patients.

In only 4/20 (20.0%) studies, a detailed clarification of the reason for APT use was reported.^18^, ^20^, ^22^, ^27^ A total of 208,595 patients with a clear indication for APT use were reported. In detail, cardiovascular diseases, cerebrovascular diseases, diabetes mellitus and arterial hypertension were reported as indication in 48.4, 35.1, 37.7 and 93.8% of the cases, respectively. As expected, in no case the indication was correlated with HCC prevention or cure.

### 3.2 Incidence of HCC with vs. without APT

According to the data shown in Table 1, 15 studies reported the incidence of HCC in patients with or without APT.^15^, ^16^, ^17^, ^18^, ^19^, ^20^, ^21^, ^22^, ^23^, ^24^, ^25^, ^26^, ^27^, ^28^, ^29^ A total of 2,685,009 patients were considered, with 25,188 (0.9%) incident HCC cases. In detail, 6328/1,238,343 (0.5%) and 18,860/1,356,666 (1.4%) new HCCs were observed in the APT and no‐APT groups, respectively.

**FIGURE 2:**
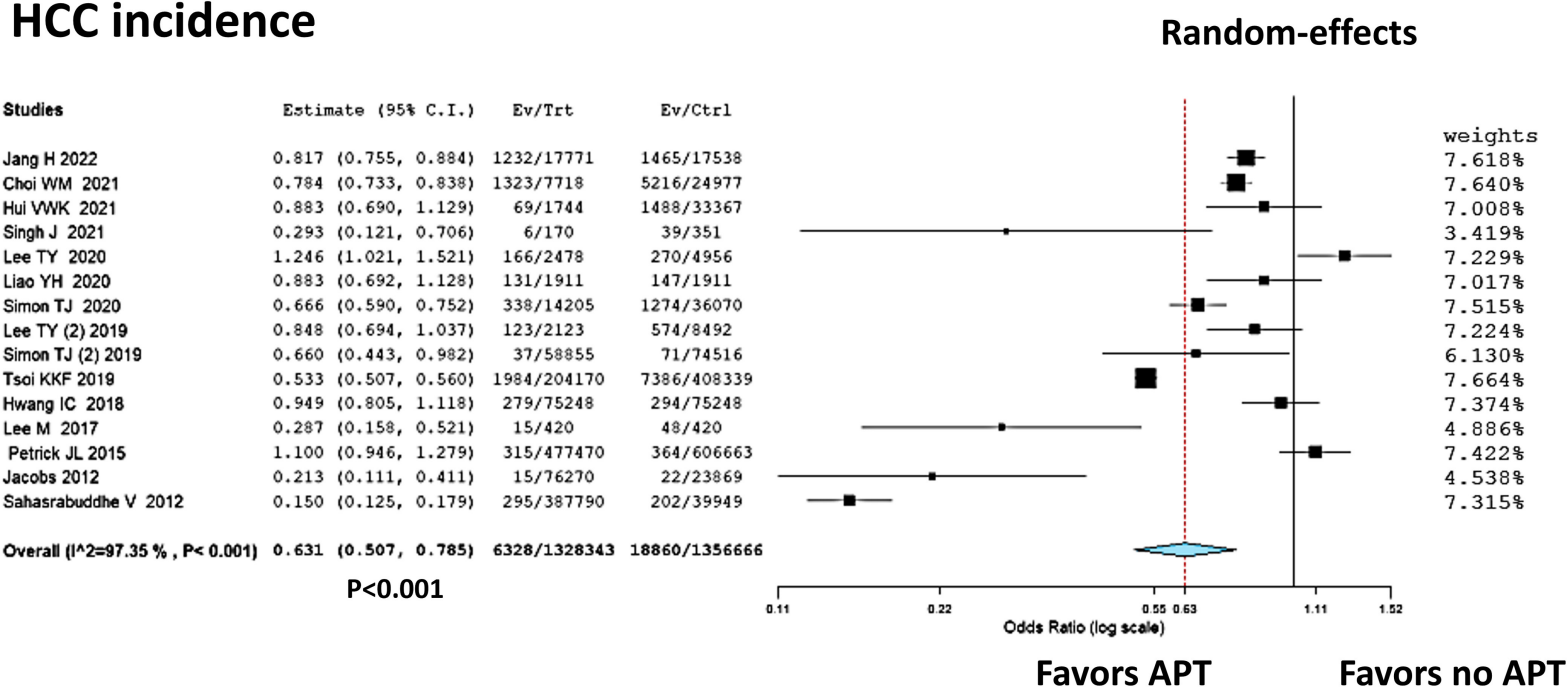
Forest plot and meta‐analysis showing the positive correlation between the use of APT and the reduced risk of HCC incidence. APT, anti‐platelet therapy; HCC, hepatocellular carcinoma

Table 2 shows a wide heterogeneity among the included studies in terms of duration of APT treatment, age, sex, proportion of patients included with a diagnosis of cirrhosis and duration of follow‐up. The meta‐analysis confirmed this heterogeneity, with an I2 = 97.4% (*p* < 0.0001). Overall, the summary OR (0.63; 95%CI, 0.51–0.79, *p* < 0.0001) showed a significant reduction in the risk of incident HCCs in cases treated with APT (Figure 2).

### 3.3 Mortality following HCC treatment with vs. without APT

According to the data shown in Table 1, 5 studies reported post‐treatment death rates in HCC patients with versus without APT.^30^, ^31^, ^32^, ^33^, ^34^ These studies included 3281 patients, with 930 (29.9%) deaths. In detail, 134/577 (23.2%) and 796/2704 (29.4%) deaths were observed in the APT and no‐APT groups, respectively. Only two studies clarified the causes of death in detail.^30^, ^33^ According to the available data, 28/102 (27.5%) and 149/322 (46.3%) HCC‐related deaths were observed in the APT and no‐APT groups, respectively. The non‐tumour‐related causes of death were 9/102 (8.8%) and 27/322 (8.4%) in the APT and no‐APT groups, respectively.

**FIGURE 3:**
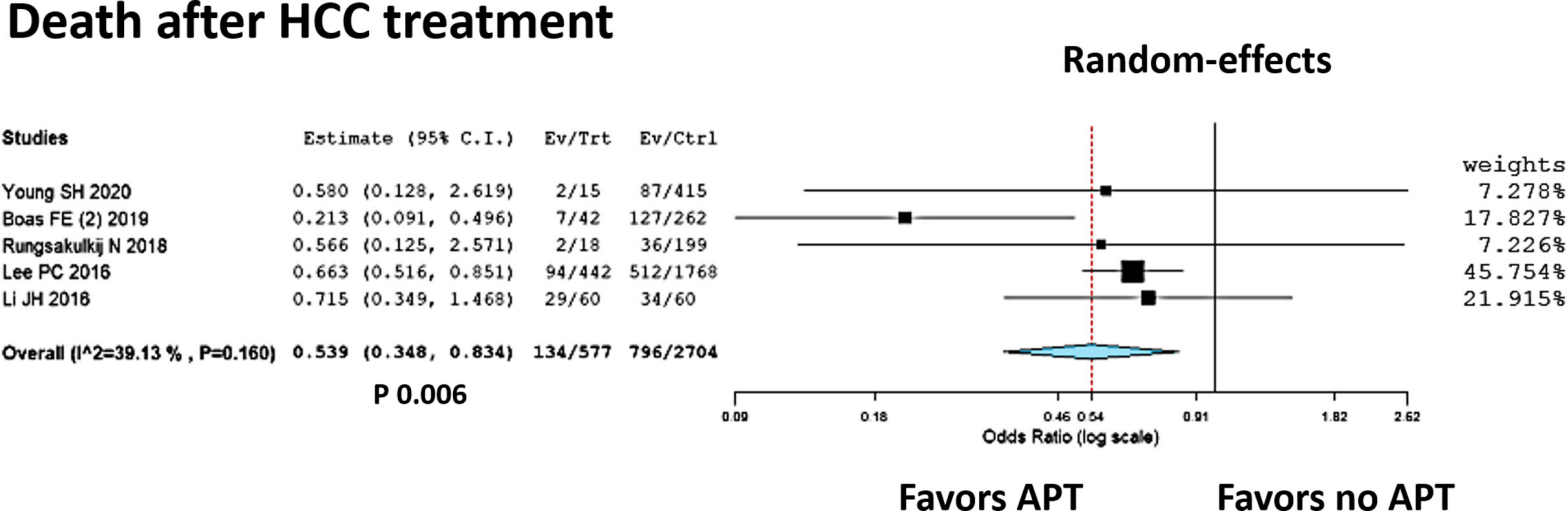
Forest plot and meta‐analysis showing the positive correlation between the use of APT and the reduced risk of overall death after any HCC treatment. APT, anti‐platelet therapy; HCC, hepatocellular carcinoma

As far as HCC treatment is concerned, a curative approach, namely surgical resection, was carried out in 3 studies.^31^, ^32^, ^34^ The remaining two studies reported a palliative approach with an intra‐arterial therapy (trans‐arterial chemoembolization or trans‐arterial embolization).^30^, ^33^ As shown in Table 2, the studies showed discrete homogeneity in age, prevalence of male sex and presence of cirrhosis. The meta‐analysis confirmed this figure with an I2 = 39.1% (*p* = 0.16). The summary OR (95%CI) showed a reduced risk of death in cases treated with APT, being 0.54 (95%CI = 0.35–0.83; *p* = 0.006) (Figure 3).

## 4 DISCUSSION

**FIGURE 4:**
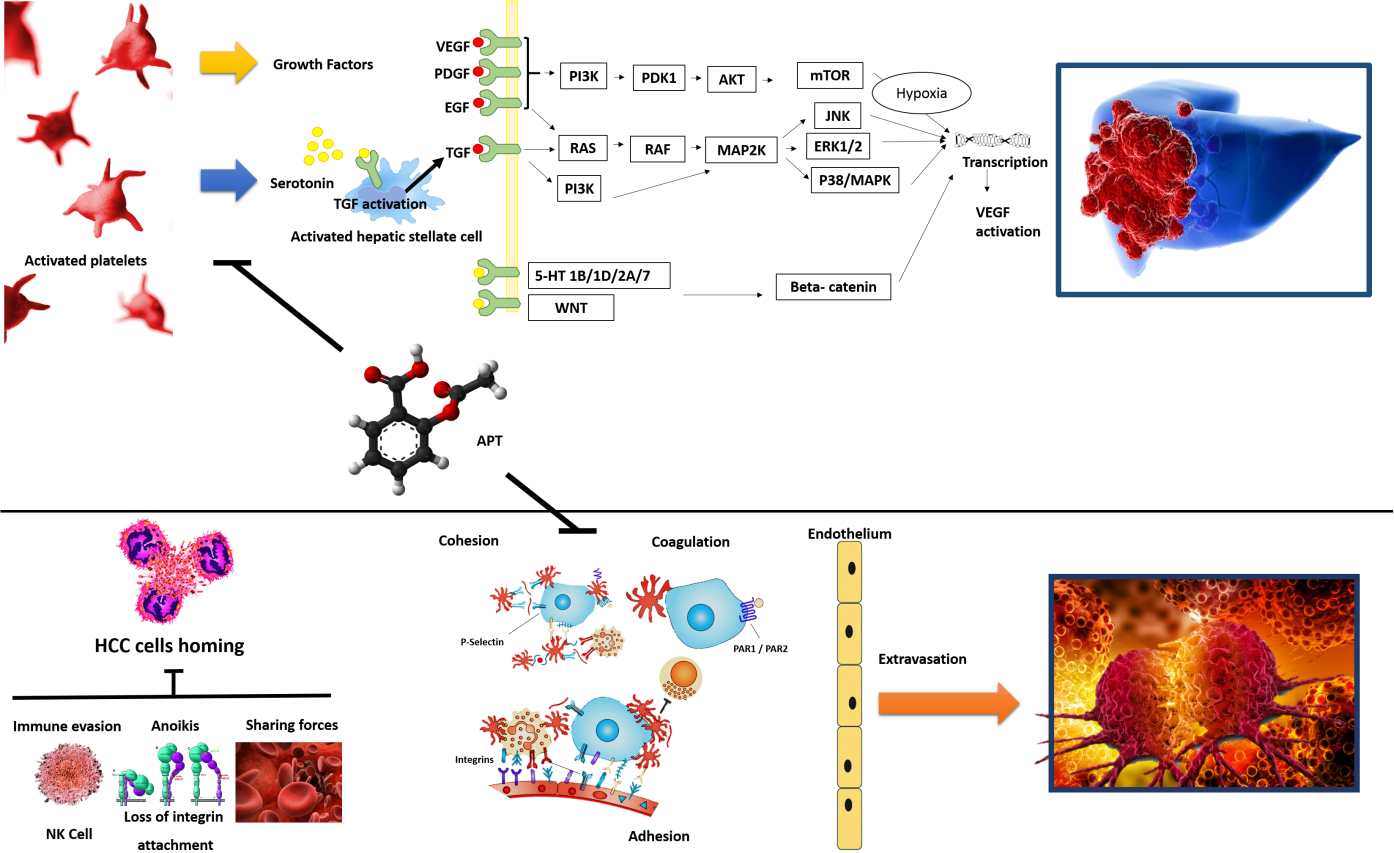
Figure reporting the mechanisms connected between platelets and HCC growth and spread, and the consequent beneficial effect of blocking these mechanisms driven by the APT. Figure modified by Lai Q, et al. (Ref. 3)

The present meta‐analysis shows a favourable relationship of APT for both the risk of HCC incidence and mortality following its treatment. This potential clinical relevance of APT in the prevention and management of HCC still represents a poorly explored field, in which the intercorrelations among platelets, carcinogenesis, tumour spread and antiplatelet therapies require a more profound comprehension (Figure 4).

The potential negative influence of platelets on HCC has been reported in numerous basic science and clinical studies, with several platelet‐related factors associated with the hepatocarcinogenesis processes.^1^ For example, serotonin plays a relevant role in tumour angiogenesis, liver regeneration and HCC growth. Serotonin receptor is overexpressed in HCC cells and can activate both the PI3K/Akt and Wnt/B‐catenin pathways signalling.^35^, ^36^ PDGF promotes liver fibrosis and hepatocarcinogenesis by activating the Ras/Raf/MEK/ERK pathway, while TGF‐β blocks the Kruppel‐like factor 6, a tumour suppressor able to repress HCC proliferation and metastatic spread.^1^EGF is a growth factor secreted by the alpha granules of the platelets, acting directly as a promoter of the inflammatory microenvironment, while VEGF is a well‐known angiogenetic factor involved in the HCC growth.^3^The relevance of these mediators is of particular interest because many of the currently available systemic therapies for HCC are based on drugs that interact directly with their activation pathways. For example, sorafenib is an anti‐PDGFR, VEGFR and EGFR agent, while regorafenib is an anti‐EGFR and anti‐VEGFR drug, and lenvatinib acts as an anti‐VEGFR.^3^

In recent years, a growing interest focused on the possible role of APT in preventing HCC, and several studies based on animal models and cell lines were published with the intent to clarify the molecular mechanisms connecting APT with the development of HCC. Sitia et al. investigated the effect of low‐dose dual APT (aspirin + clopidogrel) in an HBV‐positive transgenic mice model, observing a reduction of the intrahepatic HBV‐specific CD8+ T‐cells, a decrease in the severity of liver fibrosis and a reduced incidence of HCC.^8^, ^37^, ^38^ In this model, the anti‐cancer effect was explained by reducing immune‐mediated chronic liver injury.^1^, ^8^ Zhang et al. demonstrated in a murine model, an increased binding between platelets and poorly differentiated HCC, and the administration of clopidogrel triggered tumour cell differentiation and inhibited tumour growth.^39^ The potential mechanism associated with the preventive effect exerted by aspirin was to inhibit cell growth and induce apoptosis, involving both extrinsic and intrinsic pathways, in HepG2 tumours in BALB/c nude mice.^40^ Another mechanism by which aspirin may inhibit HCC cells growth is through decreased expression of GLUT1 and HIF1, thus leading to lowered levels of reactive oxygen species and reduced glucose consumption by tumoral cells.^41^

From the bench to the bedside, several studies conducted on humans align with the hypothesis that a higher number of platelets may be associated with worse outcomes in patients with HCC and that APT may demonstrate a protective effect.^42^, ^43^, ^44^, ^45^

The present meta‐analysis, which included 20 studies assessing the role of APT in HCC incidence and outcome following treatment, showed that patients treated with APT show a 40% reduced risk of HCC incidence and a halved risk for all‐cause mortality in patients with HCC treated with either curative or palliative strategies. Most of the studies included in the meta‐analysis derived from retrospective large national cohorts coming from Eastern countries, with a minority originating in the United States or Europe. A wide heterogeneity in the population included in the studies assessing the role of APT on the incidence of HCC has been observed, suggesting caution in the interpretation of the results. Nevertheless, a positive effect of APT was reported in most studies, with a minimum expected risk reduction of approximately 20%. The only study with contrasting results was a large Korean observational study (460,755 participants), showing the ineffectiveness of aspirin in preventing HCC in high‐risk patients, such as those with cirrhosis and elderly subjects.^19^ Conversely, in a subgroup of patients at high‐risk for HCC (i.e. viral hepatitis), aspirin (especially in association with NSAIDs) seemed to have an anti‐cancer effect. These results seem to suggest different carcinogenetic mechanisms and, at the same time, different chemo‐preventive actions of aspirin.^19^ Moreover, the available data unfortunately present a significant heterogeneity in terms of risk for HCC incidence, as in most cases low‐risk patients were enrolled in the studies. Furthermore, when high‐risk cases were explored (i.e. cirrhosis, chronic hepatitis) only patients with viral aetiology were observed. Consequently, we were not able, according with the data obtained from the meta‐analysis, to clarify the potential beneficial effect of APT in other settings (i.e. steato‐hepatitis, alcohol abuse).

While the benefit of APT on HCC incidence was demonstrated in most studies, mainly involving aspirin as APT, some of them also assessed the potential harm caused by APT treatment. In particular, the risk of bleeding was not significantly increased by aspirin alone in studies carried out in vast cohorts of patients with chronic hepatitis B in Korea and Taiwan, while the risk seemed to increase in patients treated with clopidogrel alone or with dual APT.^18^, ^22^ This absence of significant harm was also reported in a nationwide study from Sweden based on 50,275 patients with chronic hepatitis B and C, showing that the use of low‐dose aspirin was associated with a significantly lower risk of HCC and liver‐related mortality without a significantly higher risk of gastrointestinal bleeding.^23^ However, not all studies demonstrated the absence of an increased risk of bleeding with APT, as both aspirin and clopidogrel increased the risk of upper gastrointestinal bleeding in patients undergoing HCC resection, although no increased risk of major haemorrhagic events was observed in this study.^46^

As for the duration and dose of APT, some studies reported a direct correlation between them and the reduced risk of HCC incidence. A Taiwanese study showed that subjects with chronic hepatitis C had a lower cumulative incidence rate of HCC only after the first 10 years of APT treatment.^24^ A population‐based study performed on prospective cohorts of 87,507 men and 45,864 women reported an aspirin dose‐dependent and duration‐dependent reduction in HCC risk that was similar in patients with and without cirrhosis. In contrast, no significant HCC risk reduction was observed in patients regularly assuming NSAIDs.^21^ A study from Sweden based on 50,275 cases reported a duration‐dependent association between HCC incidence and APT. Compared with short‐term APT use (3 months to <1 year), a progressive reduction of the risk in HCC incidence was observed after 1–3 years (adjusted hazard ratio = 0.90, 95%CI = 0.76–1.06), after 3–5 years (adjusted hazard ratio = 0.66; 95%CI = 0.56–0.78) and >5 years (adjusted hazard ratio = 0.57, 95%CI = 0.42–0.70) of APT use.^23^ Unfortunately, due to the great heterogeneity reported in the selected studies, we were not able to perform sub‐analyses focused on the concept of APT dosage and duration. However, although it is not possible to have a firm position on this topic, it looks suggestive to consider that the positive effect of APT should be conditioned by the time of administration and dosage, and that this aspect surely requires further investigations with the intent to address solid recommendations on the management of patients at high risk for HCC.

As far as the outcome following HCC treatment is concerned, the positive effect of APT in terms of mortality after HCC treatment was reported in studies focused on curative and non‐curative strategies.

In studies assessing curative approaches, two experiences from Taiwan carried out in patients with hepatitis B‐related HCC treated with liver resection reported better overall survival rates in patients receiving APT, independently of the concomitant treatment with non‐steroidal anti‐inflammatory drugs (NSAIDs).^31^, ^34^

On the other hand, considering non‐curative approaches, a study from the United States focused on the use of trans‐arterial embolization reported an association between aspirin use and improved liver function test results and survival (median survival period after initial embolization: 57 vs. 23 months; *p* = 0.008).^33^

Unfortunately, also in this case, it was impossible to perform a solid sub‐analysis splitting the data of curative and non‐curative approaches. We can only postulate that the use of APT should relate to different ranges of survivals according to the initial stage of HCC and the efficacy of the therapy used, although we feel that also in this setting more studies are needed to better explore this relevant issue.

It was also impossible to clarify if the main cause of death in the investigated patients was tumour‐related. This limit derived from the small number of studies specifically detailing the causes of death.^30^, ^33^ However, it was interesting to note that, only looking at these studies, the number of tumour‐unrelated deaths was similar between APT and no‐APT groups (8.8 vs. 8.4%), while the number of HCC‐related deaths was significantly higher in the no‐APT group (46.3 vs. 27.5%).

Our study also has some limitations. First, the number of studies available for the analysis is limited, and only five studies assessed the potential association of APT with the outcome of HCC patients following cancer treatment. Unfortunately, the limited number of cases to explore determined our decision to focus only on two endpoints commonly reported in the studies, namely incidence of HCC and post‐treatment death, avoiding exploring other less reported aspects like the incidence of clinically relevant bleeding in patients treated with APT. Secondly, several relevant aspects like the effective duration of APT therapy, its doses, the indications for APT administration and treatment compliance are all potential confounders that were impossible to thoroughly investigate due to the lack of these data in most of the original studies. Moreover, we should also underline that every trial that investigates the use of aspirin is prone to external contamination since, at least in Western countries, patients can easily access to this drug even outside the trial.

Thirdly, the small number of studies assessing outcomes following HCC treatment limited our ability to perform sub‐analyses focused on the association between APT use and type of treatment, tumour burden, and cause of underlying liver disease.

Lastly, another relevant aspect that should be considered is the interaction of APT with other concomitant drugs that may potentially impact HCC prevention, like antiviral agents, anti‐diabetic medications and statins.^47^, ^48^, ^49^

In conclusion, the results obtained from this meta‐analysis suggest a correlation between APT and cancer development exist. APT may be considered a promising agent able to modulate the risk of HCC and improve post‐treatment overall outcomes. Among antiplatelet drugs, aspirin seems to be the agent that reportedly had the most relevant favourable effect. The potential efficacy and safety of APT regarding the risk of gastrointestinal bleeding needs to be assessed in larger studies.

## AUTHOR CONTRIBUTIONS

QL and EGG contributed to the conception and design of the study; QL, NDM, GM, FMe and RP contributed to the acquisition of data; QL analysed and interpreted the data; QL, GG, FM and EGG drafted the article; MF, DN and EGG critically revised the manuscript; all authors approved the final version.

## FUNDING INFORMATION

No specific funds were received to carry out this study.

## CONFLICT OF INTEREST

The authors have no conflicts of interest to declare about the present study.

## APPENDIX A

### Collaborators of the Associazione Italiana per lo Studio del Fegato (AISF) HCC Special Interest Group

Aglitti A, Aliberti C, Baccarani U, Bhoori S, Borzio M, Brancaccio G, Burra P, Cabibbo G, Casadei Gardini A, Carrai P, Cillo U, Conti F, Cucchetti A, D'Ambrosio R, Dell'Unto C, Di Costanzo GG, Di Sandro S, Foschi FG, Fucilli F, Gambato M, Gasbarrini A, Giuliante F, Ghinolfi D, Grieco A, Gruttaduria S, Guarino M, Kostandini A, Iavarone M, Lenci I, Levi Sandri GB, Losito F, Lupo LG, Manzia TM, Mazzocato S, Mescoli C, Miele L, Muley M, Persico M, Plaz Torres MC, Pompili M, Ponziani FR, Rapaccini GL, Rendina M, Renzulli M, Rossi M, Rreka E, Russo FP, Sacco R, Sangiovanni A, Sessa A, Simonetti N, Sposito C, Tortora R, Trevisani F, Viganò L, Viganò M, Villa E, Vincenzi V, Violi P, Vitale A.

## References

[1] Pavlovic N , Rani B , Gerwins P , Heindryckx F . Platelets as key factors in hepatocellular carcinoma. Cancers (Basel). 2019;11(7):1022. 31330817 10.3390/cancers11071022 PMC6678690

[2] Carr BI , Guerra V , Giannini EG , et al. Italian liver cancer group. Significance of platelet and AFP levels and liver function parameters for HCC size and survival. Int J BiolMarkers. 2014;29(3):e215‐e223. doi:10.5301/jbm.5000064 24526315

[3] Lai Q , Vitale A , Manzia TM , et al. Platelets and hepatocellular cancer: bridging the bench to the clinics. Cancers (Basel). 2019;11(10):1568. 31618961 10.3390/cancers11101568 PMC6826649

[4] Bibbins‐Domingo K , Force USPST . Aspirin use for the primary prevention of cardiovascular disease and colorectal cancer: U.S. preventive services task force recommendation statement. Ann Intern Med. 2016;164:836‐845. 27064677 10.7326/M16-0577

[5] Hayashi T , Shibata M , Oe S , Miyagawa K , Honma Y , Harada M . Antiplatelet therapy improves the prognosis of patients with hepatocellular carcinoma. Cancers (Basel). 2020;12(11):3215. doi:10.3390/cancers12113215 33142758 PMC7693153

[6] Casadei‐Gardini A , Rovesti G , Dadduzio V , et al. Impact of aspirin on clinical outcome in advanced HCC patients receiving sorafenib and regorafenib. HPB (Oxford). 2021;23(6):915‐920. doi:10.1016/j.hpb.2020.09.024 Epub 2020 Nov 12. 33191108

[7] Ielasi L , Tovoli F , Tonnini M , et al. Beneficial prognostic effects of aspirin in patients receiving sorafenib for hepatocellular carcinoma: a tale of multiple confounders. Cancers (Basel). 2021;13(24):6376. doi:10.3390/cancers13246376 34944996 PMC8699252

[8] Aiolfi R , Sitia G . Chronic hepatitis B: role of antiplatelet therapy in inflammation control. Cell Mol Immunol. 2015;12(3):264‐268. 25578311 10.1038/cmi.2014.124 PMC4654314

[9] Lucotti S , Cerutti C , Soyer M , et al. Aspirin blocks formation of metastatic intravascular niches by inhibiting platelet‐derived COX‐1/thromboxane A2. J Clin Invest. 2019;129(5):1845‐1862. 30907747 10.1172/JCI121985 PMC6486338

[10] Ramadori P , Klag T , Malek NP , Heikenwalder M . Platelets in chronic liver disease, from bench to bedside. JHEP. 2019;1(6):448‐459. 10.1016/j.jhepr.2019.10.001 PMC7005648 32039397

[11] Smyth SS , Reis ED , Zhang W , Fallon JT , Gordon RE , Coller BS . Beta(3)‐integrin‐deficient mice but not P‐selectin‐deficient mice develop intimal hyperplasia after vascular injury: correlation with leukocyte recruitment to adherent platelets 1 hour after injury. Circulation. 2001;103(20):2501‐2507. 11369692 10.1161/01.cir.103.20.2501

[12] Pavlović N , Kopsida M , Gerwins P , Heindryckx F . Activated platelets contribute to the progression of hepatocellular carcinoma by altering the tumor environment. Life Sci. 2021;277:119612. 33991548 10.1016/j.lfs.2021.119612

[13] Hutton B , Salanti G , Caldwell DM , et al. The PRISMA extension statement for reporting of systematic reviews incorporating network meta‐analyses of health care interventions: checklist and explanations. Ann Intern Med. 2015;162(11):777‐784. doi:10.7326/M14-2385 26030634

[14] Stang A . Critical evaluation of the Newcastle‐Ottawa scale for the assessment of the quality of nonrandomized studies in meta‐analyses. Eur J Epidemiol. 2010;25:603‐605. doi:10.1007/s10654-010-9491-z 20652370

[15] Sahasrabuddhe VV , Gunja MZ , Graubard BI , et al. Non‐steroidal anti‐inflammatory drug use, chronic liver disease, and hepatocellular carcinoma. J Natl Cancer Inst. 2012;104(23):1808‐1814. doi:10.1093/jnci/djs452 23197492 PMC3514167

[16] Jacobs EJ , Newton CC , Gapstur SM , Thun MJ . Daily aspirin use and cancer mortality in a large US cohort. J Natl Cancer Inst. 2012;104(16):1208‐1217. doi:10.1093/jnci/djs318 22888140

[17] Petrick JL , Sahasrabuddhe VV , Chan AT , et al. NSAID use and risk of hepatocellular carcinoma and intrahepatic cholangiocarcinoma: the liver cancer pooling project. Cancer Prev Res (Phila). 2015;8(12):1156‐1162. doi:10.1158/1940-6207.CAPR-15-0126 26391917 PMC4704448

[18] Lee M , Chung GE , Lee JH , et al. Antiplatelet therapy and the risk of hepatocellular carcinoma in chronic hepatitis B patients on antiviral treatment. Hepatology. 2017;66(5):1556‐1569. doi:10.1002/hep.29318 28617992

[19] Hwang IC , Chang J , Kim K , Park SM . Aspirin use and risk of hepatocellular carcinoma in a National Cohort Study of Korean adults. Sci Rep. 2018;8(1):4968. doi:10.1038/s41598-018-23343-0 29563592 PMC5862896

[20] Lee TY , Hsu YC , Tseng HC , et al. Association of daily aspirin therapy with risk of hepatocellular carcinoma in patients with chronic hepatitis B. JAMA Intern Med. 2019;179(5):633‐640. doi:10.1001/jamainternmed.2018.8342 30882847 PMC6503573

[21] Simon TG , Ma Y , Ludvigsson JF , et al. Association between aspirin use and risk of hepatocellular carcinoma. JAMA Oncol. 2018;4(12):1683‐1690. doi:10.1001/jamaoncol.2018.4154 Erratum in: JAMA Oncol 2019 Apr 1;5(4):579. PMID: 30286235; PMCID: PMC6440745. 30286235 PMC6440745

[22] Tsoi KKF , Ho JMW , Chan FCH , Sung JJY . Long‐term use of low‐dose aspirin for cancer prevention: a 10‐year population cohort study in Hong Kong. Int J Cancer. 2019;145(1):267‐273. doi:10.1002/ijc.32083 30575949

[23] Lee TY , Hsu YC , Tseng HC , Lin JT , Wu MS , Wu CY . Association of daily aspirin therapy with hepatocellular carcinoma risk in patients with chronic hepatitis C virus infection. Clin Gastroenterol Hepatol. 2020;18(12):2784‐2792. doi:10.1016/j.cgh.2020.04.036 32360983

[24] Liao YH , Hsu RJ , Wang TH , et al. Aspirin decreases hepatocellular carcinoma risk in hepatitis C virus carriers: a Nationwide cohort study. BMC Gastroenterol. 2020;20(1):6. doi:10.1186/s12876-020-1158-y 31918672 PMC6953130

[25] Simon TG , Duberg AS , Aleman S , Chung RT , Chan AT , Ludvigsson JF . Association of aspirin with hepatocellular carcinoma and liver‐related mortality. N Engl J Med. 2020;382(11):1018‐1028. doi:10.1056/nejmoa1912035 32160663 PMC7317648

[26] Choi WM , Kim HJ , Jo AJ , et al. Association of aspirin and statin use with the risk of liver cancer in chronic hepatitis B: a nationwide population‐based study. Liver Int. 2021;41:2777‐2785. doi:10.1111/liv.15011 34242482

[27] Hui VW , Yip TC , Wong VW , et al. Aspirin reduces the incidence of hepatocellular carcinoma in patients with chronic hepatitis B receiving Oral Nucleos(t)ide analog. ClinTranslGastroenterol. 2021;12(3):e00324. doi:10.14309/ctg.0000000000000324 PMC8126482 33750746

[28] Singh J , Wozniak A , Cotler SJ , et al. Combined use of aspirin and statin is associated with a decreased incidence of hepatocellular carcinoma. J Clin Gastroenterol. 2021;56:369‐373. doi:10.1097/MCG.0000000000001546 33883511

[29] Jang H , Lee YB , Moon H , et al. Aspirin use and risk of hepatocellular carcinoma in patients with chronic hepatitis B with or without cirrhosis. Hepatology. 2022;76:492‐501. doi:10.1002/hep.32380 35100447

[30] Li JH , Wang Y , Xie XY , et al. Aspirin in combination with TACE in treatment of unresectable HCC: a matched‐pairs analysis. Am J Cancer Res. 2016;6(9):2109‐2116. 27725915 PMC5043119

[31] Lee PC , Yeh CM , Hu YW , et al. Antiplatelet therapy is associated with a better prognosis for patients with hepatitis B virus‐related hepatocellular carcinoma after liver resection. Ann Surg Oncol. 2016;23(Suppl 5):874‐883. doi:10.1245/s10434-016-5520-9 27541812

[32] Rungsakulkij N , Suragul W , Mingphruedhi S , Tangtawee P , Muangkaew P , Aeesoa S . Prognostic factors in patients withHBV‐Rrelated hepatocellular carcinoma following hepatic resec‐tion. Infect Agent Cancer. 2018;13:20. 29930697 10.1186/s13027-018-0192-7 PMC5994073

[33] Boas FE , Brown KT , Ziv E , et al. Aspirin is associated with improved liver function after embolization of hepatocellular carcinoma. AJR Am J Roentgenol. 2019;213(3):1‐7. doi:10.2214/AJR.18.20846 Epub 2019 May 23. Erratum in: AJR Am J Roentgenol. 2019 Nov;213(5):1174. PMID: 31120783; PMCID: PMC6709849. PMC6709849 31120783

[34] Young SH , Chau GY , Lee IC , et al. Aspirin is associated with low recurrent risk in hepatitis B virus‐related hepatocellular carcinoma patients after curative resection. J Formos Med Assoc. 2020;119(1 Pt 2):218‐229. doi:10.1016/j.jfma.2019.04.018 31104872

[35] Zuo X , Chen Z , Cai J , et al. 5‐Hydroxytryptamine receptor 1D aggravates hepatocellular carcinoma progression through FoxO6 in AKT‐dependent and independent manners. Hepatology. 2019;69(5):2031‐2047. 30561038 10.1002/hep.30430

[36] Fatima S , Shi X , Lin Z , et al. 5‐Hydroxytryptamine promotes hepatocellular carcinoma proliferation by influencing β‐catenin. Mol Oncol. 2016;10(2):195‐212. 26474915 10.1016/j.molonc.2015.09.008 PMC5528951

[37] Sitia G , Aiolfi R , Di Lucia P , et al. Antiplatelet therapy prevents hepatocellular carcinoma and improves survival in a mouse model of chronic hepatitis B. Proc Natl Acad Sci U S A. 2012;109(32):E2165‐E2172. 22753481 10.1073/pnas.1209182109 PMC3420192

[38] Sitia G , Iannacone M , Guidotti LG . Antiplatelet therapy in the prevention of hepatitis B virus‐associated hepatocellular carcinoma. J Hepatol. 2013;59(5):1135‐1138. 23742914 10.1016/j.jhep.2013.05.040

[39] Zhang R , Guo H , Xu J , et al. Activated platelets inhibit hepatocellular carcinoma cell differentiation and promote tumor progression via platelet‐tumor cell binding. Oncotarget. 2016;7(37):60609‐60622. 27542264 10.18632/oncotarget.11300 PMC5312405

[40] Hossain MA , Kim DH , Jang JY , et al. Aspirin induces apoptosis in vitro and inhibits tumor growth of human hepatocellular carcinoma cells in a nude mouse xenograft model. Int J Oncol. 2012;40(4):1298‐1304. 22179060 10.3892/ijo.2011.1304 PMC3584583

[41] Liu Y‐X , Feng J‐Y , Sun M‐M , et al. Aspirin inhibits the proliferation of hepatoma cells through controlling GLUT1‐mediated glucose metabolism. Acta Pharmacol Sin. 2019;40(1):122‐132. 29925918 10.1038/s41401-018-0014-x PMC6318307

[42] Pravisani R , Mocchegiani F , Isola M , et al. Postoperative trends and prognostic values of inflammatory and nutritional biomarkers after liver transplantation for hepatocellular carcinoma. Cancers (Basel). 2021;13(3):513. doi:10.3390/cancers13030513 33572776 PMC7866292

[43] Lai Q , Melandro F , LarghiLaureiro Z , et al. Platelet‐to‐lymphocyte ratio in the setting of liver transplantation for hepatocellular cancer: a systematic review and meta‐analysis. World J Gastroenterol. 2018;24(15):1658‐1665. doi:10.3748/wjg.v24.i15.1658 29686473 PMC5910549

[44] Tan RZH , Lockart I , Abdel Shaheed C , Danta M . Systematic review with meta‐ analysis: the effects of non‐steroidal anti‐inflammatory drugs and antiplatelet therapy on the incidence and recurrence of hepatocellular carcinoma. Aliment Pharmacol Ther. 2021;54(4):356‐367. doi:10.1111/apt.16515 34247393

[45] Li X , Yu Y , Liu L . Influence of aspirin use on clinical outcomes of patients with hepatocellular carcinoma: a meta‐analysis. Clin Res Hepatol Gastroenterol. 2021;45(6):101545. doi:10.1016/j.clinre.2020.09.006 33067170

[46] Lee PC , Yeh CM , Hu YW , et al. Antiplatelet therapy is associated with a better prognosis for patients with hepatitis B virus‐related hepatocellular carcinoma after liver resection. Ann Surg Oncol. 2016;23(Suppl 5):874‐883. 27541812 10.1245/s10434-016-5520-9

[47] Choi WM , Choi J , Lim YS . Effects of tenofovir vs entecavir on risk of hepatocellular carcinoma in patients with chronic HBV infection: a systematic review and Metaanalysis. Clin Gastroenterol Hepatol. 2021;19(2):246‐258. doi:10.1016/j.cgh.2020.05.008 32407970

[48] Plaz Torres MC , Jaffe A , Perry R , Marabotto E , Strazzabosco M , Giannini EG . Diabetes medications and risk of HCC. 2022. doi:10.1002/hep.32439 PMC9790535 35239194

[49] Singh S , Singh PP , Singh AG , Murad MH , Sanchez W . Statins are associated with a reduced risk of hepatocellular cancer: a systematic review and meta‐analysis. Gastroenterology. 2013;144(2):323‐332. doi:10.1053/j.gastro.2012.10.005 23063971

